# Case fatality ratio of COVID-19 patients requiring invasive mechanical ventilation in Mexico: an analysis of nationwide data

**DOI:** 10.1186/s13054-021-03485-w

**Published:** 2021-02-16

**Authors:** Silvio A. Ñamendys-Silva

**Affiliations:** 1 Division of Pulmonary, Anesthesia and Critical Care Medicine, Instituto Nacional de Ciencias Médicas y Nutrición Salvador Zubirán, 14000 Mexico City, Mexico; 2 Department of Critical Care Medicine, Instituto Nacional de Cancerología, Mexico City, Mexico; 3 Society of Physicians of Medica Sur (Member), Mexico City, Mexico

As of December 15, 2020, a total of 70,476,836 confirmed cases of coronavirus disease 2019 (COVID-19) had been reported to the World Health Organization, along with 1,599,922 deaths [1]. The demand for hospital and intensive care unit (ICU) beds and resources to care for COVID-19 patients has been significant worldwide. The aim of the present report is to describe the case fatality ratio (CFR) of COVID-19 patients requiring invasive mechanical ventilation in Mexico.

This report analyses of an anonymized patient dataset that is publicly available and accessible to anyone through the Mexican Health Ministry and that was released on December 15, 2020 [2]. All patients with laboratory-confirmed severe acute respiratory syndrome coronavirus 2 infection according to positive reverse transcriptase-polymerase chain reaction approved by adjudication committees or epidemiological associations were included.

The CFR for COVID-19 was calculated as the total number of deaths due to COVID-19 divided by the number of total confirmed COVID-19 cases as of December 15, 2020, multiplied by 100 [3]. A total of 12,018 mechanically ventilated adults with COVID-19 from a previous report were included in the present analysis [4].

Between February 28 and December 15, 2020, a total of 1,267,202 confirmed cases of COVID-19 were reported in Mexico. The sociodemographic characteristics and comorbidities of all patients with COVID-19 in Mexico are shown in Table 1. A total of 115,099 deaths occurred, for an overall CFR of 9.1%. A total of 15.3% (39,848/260,859) of hospitalized patients required invasive mechanical ventilation (IMV), and 70.8% (28,209/39,848) of those patients received IMV outside the ICU (Table 1). Overall, the CRF was higher in patients mechanically ventilated in the ward (outside the ICU) (23,823 [84.5%] of 28,209) than in those admitted to the ICU (8433 [72.5%] of 11,639, *p* < 0.001). Figure 1 shows the epidemiological  curve of the invasively ventilated patients with confirmed cases of COVID-19 (survivor and nonsurvivors) plotted by hospital admission date.

**Table 1:** Patients, deaths, and case fatality ratio for the 1,267,202 confirmed COVID-19 patients in Mexico as of December 15, 2020

Baseline characteristics	Confirmed cases, N (%)	Deaths, N (%)	Case fatality ratio, %
Overall	1,267,202	115,099	9.1
Age, years			
0–20	69,976 (5.5)	519 (0.5)	0.74
21–40	506,113 (39.9)	6651 (5.7)	1.31
41–50	262,871 (20.7)	13,963 (12.1)	5.3
51–60	210,113 (16.6)	25,948 (22.5)	12.3
61–70	126,797 (10.0)	32,154 (27.9)	25.4
71–80	64,912 (5.1)	24,214 (21.0)	37.3
81–90	23,217 (1.8)	10,313 (8.9)	44.4
> 90	3203 (0.3)	1337 (1.16)	41.7
Sex			
Female	626,096 (49.4)	42,011(36.5)	6.7
Male	641,106 (50.6)	73,088 (63.5)	11.4
Comorbidities			
Hypertension	231,328 (18.2)	52,593 (45.6)	22.7
Obesity	205,652 (16.2)	27,089 (23.5)	13.2
Diabetes	180,165 (14.2)	44,367 (38.6)	24.6
Cardiovascular disease	22,343 (1.8)	6141 (5.3)	27.5
Chronic kidney disease	21,363 (1.7)	8445 (7.3)	39.5
Chronic obstructive lung disease	15,945 (1.3)	5460 (4.7)	34.2
Immunosuppression	12,180 (0.9)	2792 (2.4)	22.9
Medical treatment in the public healthcare system	1,231,245 (97.2)	113,311 (98.4)	9.2
Medical treatment in the private healthcare system	35,957 (2.8)	1788 (1.6)	5.1
Outpatients	1,006,343 (79.4)	12,111 (10.5)	1.2
Inpatients	260,859 (20.6)	102,988 (89.5)	39.5
Patients requiring intubation and mechanical ventilation	39,848 (15.3)	32,256 (31.2)	80.9
In the intensive care unit	11,639 (29.2)	8433 (26.1)	72.5
Outside of the intensive care unit	28,209 (70.8)	23,823 (73.9)	84.5
Patients who were not intubated but receiving oxygen therapy	221,011 (84.7)	70,732 (68.7)	32.0

**Fig. 1:**
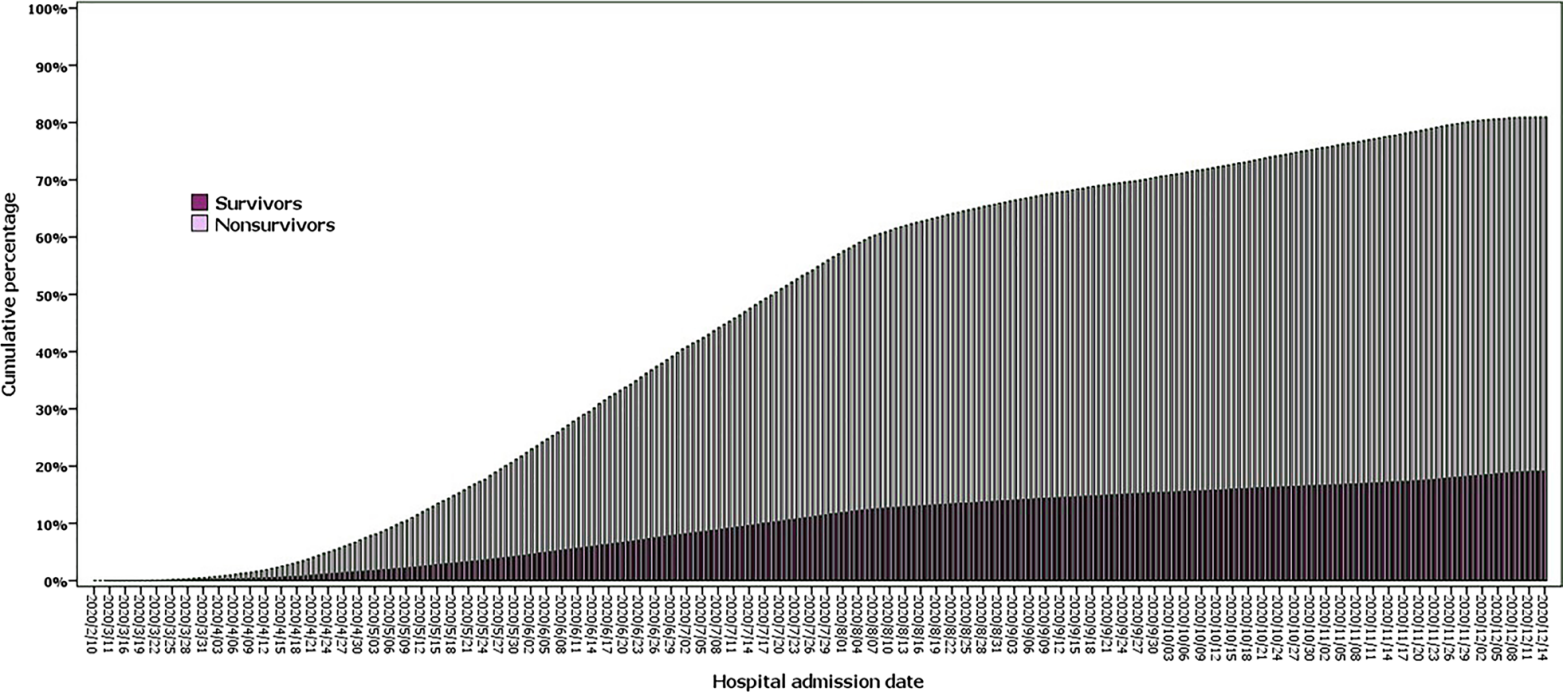
Epidemiological curve of the invasively ventilated patients with confirmed cases of COVID-19 (survivor and nonsurvivors) plotted by hospital admission date

CRF was highest in patients needing intubation and IMV, especially in a setting outside the ICU. The CFR of COVID-19 patients requiring IMV varies greatly between countries; Mexico´s CFR for COVID-19 patients requiring IMV is higher than Brazil´s (80.9% [95% confidence interval, 80.0–81.8] versus 79.7% [95% confidence interval, 78.9–80.5], *p* = 0.0497) [5]. The COVID-19 pandemic is placing unprecedented demands on Mexico’s entire health care system. Over the past 10 months, the number of ICU beds or "beds with ventilators" in Mexico has increased from 2446 to 11,634 [6]. Mexican health authorities have stated that the response to the pandemic has been satisfactory because sufficient number of "beds with ventilators" are available [4]. However, having open "beds with ventilators" in hospital wards does not mean that hospitals are equipped to handle more critically ill COVID-19 patients, which can cause a worsening of the quality of care (CFR can rise). Healthcare systems should be concerned about having sufficient qualified personnel and equipment in hospital wards, which has been one of the main problems worldwide during the COVID-19 pandemic. Although the treatment of patients in the ICU has improved in recent years, the standard of care for critically ill COVID-19 patients outside of the ICU is controversial. Daily ward rounds are usually led by an intensivist or critical care nurse (she or he) to explore the critical events for each patient, possible solutions, and prioritization of treatment. This approach can contribute to improved care and decreased CFR in a setting outside of the ICU.

Finally, although this study used nationwide data, administrative data are a source of information regarding real world clinical practices across geographic regions and health systems during the COVID-19 pandemic.

## Data Availability

http://datosabiertos.salud.gob.mx/gobmx/salud/datos_abiertos/historicos/12/datos_abiertos_covid19_15.12.2020.zip (accessed December 16, 2020).
